# Two new species of the genus *Eremiothrips* Priesner (Thysanoptera, Thripidae) from Saudi Arabia

**DOI:** 10.3897/zookeys.1291.200571

**Published:** 2026-09-11

**Authors:** Iftekhar Rasool, Hathal M. Aldhafer

**Affiliations:** 1 Department of Plant Protection, College of Food and Agriculture Sciences, King Saud University, P.O. Box 2460, Riyadh 11451, Saudi Arabia Department of Plant Protection, College of Food and Agriculture Sciences, King Saud University Riyadh Saudi Arabia

**Keywords:** drepanae, habitat association, key, male morphology, sternal pore plates, thrips, xeric habitat

## Abstract

The genus *Eremiothrips* Priesner, which inhabits xeric environments, is studied. The male of *E. brunneus* (zur Strassen) is described for the first time. Two new species, *E. tetraenae***sp. nov**. collected from *Tetraena simplex* and *E. xerophilus***sp. nov**. from *Tamarix* sp., are also described. A key to the *Eremiothrips* species lacking drepanae on abdominal tergite IX is provided.

## Introduction

The genus *Eremiothrips* Priesner is a delicate, eremophilous genus that is primarily associated with xeric environments. It comprises 22 described species that are distributed from Morocco through the Sahara Desert to the Arabian Peninsula and Iran, with a few species also found in northern Mediterranean regions, India and China (Minaei and Şahin Negiş 2026). Nine of these species are known to occur in Saudi Arabia and the Arabian Peninsula prior to the present study (Rasool et al. 2021, 2023), and three additional species are added in the current study. The genus has often been regarded as predominantly a Mediterranean inhabitant (Minaei and Şahin Negiş 2026). However, the presence of a large number of species in the hyper-arid climate of the Sahara Desert, the world’s largest continuous sand sea, “the Empty Quarter” (the Rub’ al-Khali) in the Arabian Peninsula, indicates a substantial Saharo-Arabian component. The members of this genus are generally very small and usually quite pale in color. Therefore, the somewhat smaller and considerably rarer males are often too easily overlooked (zur Strassen 1975). These males exhibit several distinctive characters on abdominal segment IX and sternites III–VII that are very helpful in species recognition. The males of *E. brunneus* (zur Strassen) and *E. imitator* Priesner were previously unknown, but the male of *E. brunneus* is described here for the first time. However, the male of *E. imitator* remains unknown; females of the species are distinguished based on simple sense cones on antennal segments III–IV (Bhatti et al. 2003). Hitherto, males of only four *Eremiothrips* species (*E. bhattii* Minaei, *E. farsi* Bhatti & Telmadarraiy, *E. manolachei* (Knechtel), and *E. similis* Bhatti) were known to lack paired drepanae on abdominal segment IX (Bhatti et al. 2003; Minaei 2012), though *E. similis* has hardly visible, greatly reduced, colorless, sclerotized triangular-shaped angles on abdominal tergites IX (Ramezani et al. 2009) that give an impression of drepanae. However, the male of *E. brunneus*, along with *E. tetraenae* sp. nov. and *E. xerophilus* sp. nov. described here, are further members of the genus that lack such paired drepanae on segment IX. Although members of the genus are considered phytophagous, their distribution depends largely on the particular habitat rather than specific host plant (Minaei 2012). In Saudi Arabia, these species have frequently been collected from succulent plants of the families Amaranthaceae, Cleomaceae, Poaceae, Zygophyllaceae as well as from trees of Tamaricaceae adapted to the xeric ecosystem.

The aims of this work are to describe two new species, describe the male of *E. brunneus* for the first time and to present a key to seven species that lack paired drepanae.

## Material and methods

The specimens were collected from southwestern, northern and central regions of Saudi Arabia by beating (BT) the plants directly into 80% ethanol vials. Mound and Marullo (1996) were followed to prepare the microscopic slides. Morphological structures were examined using a Leica DM2500 phase-contrast microscope. Images were produced using a Syncroscopy Automontage system attached to the microscope. All the measurements were taken in microns. The paratype of *E. brunneus* bearing catalog number NHMUK014232449 was examined by Dan Hall, curator of small orders at the Natural History Museum (**NHM**), London, to confirm the placement of the S1 setae on sternum VII. All specimens were deposited in the collection of King Saud University Museum of Arthropods (**KSMA**), Department of Plant Protection, College of Food and Agriculture Sciences, King Saud University.

## Results

### Key to the male *Eremiothrips* species that lack paired drepanae

**Table:** 

1	Antennae 8-segmented; pronotum with two pairs of posteroangular setae; abdominal sternites III–VII with oval to transverse pore plates	***E. bhattii***
–	Antennae 9-segmented; pronotum with one pair of posteroangular setae (Figs 7, 16); abdominal sternites with pore plates different (Figs 3, 6, 13, 23)	**2**
2	Abdominal sternites with pore plates on segments IV–VII (Fig. 3) or VI–VII (Fig. 6)	**3**
–	Abdominal sternites with pore plates on segments III–VII (Figs 13, 23)	**6**
3	Pore plates present on abdominal sternites VI–VII (Fig. 6)	***E. similis***
–	Pore plates present on abdominal sternites IV–VII	**4**
4	Head with three pairs of anteocellar setae (Fig. 1); pore plates large and transverse (Fig. 3); [in females, setae S1 on sternum VII arise at or near to posterior margin (Fig. 2), body color usually grey]	***E. brunneus***
–	Head with two pairs of anteocellar setae; pore plates transverse or oval; [in females, setae S1 on sternum VII arise far ahead of the posterior margin, body color largely pale]	**5**
5	Ocellar setae pair III well developed, distinctly longer than the distance between their bases and diameter of ocelli; pore plates transverse (Fig. cf. 3)	***E. manolachei***
–	Ocellar setae pair III minute, shorter than the distance between their bases and diameter of ocelli; pore plates oval	***E. farsi***
6	Ocellar setae pair III minute, scarcely as long as longitudinal diameter of ocellus, distinctly shorter than the distance between their bases (Fig. 7); pore plates strongly developed, longer than the basal distance between posteromarginal setae S1 (Fig. 13); [abdominal tergites in females with lateral grey patch (Fig. 12)]	***E. tetraenae* sp. nov**.
–	Ocellar setae pair III long, distinctly longer than ocellus, longer or as long as the distance between their bases (Fig. 16); pore plates hardly visible, shorter than the basal distance between posteromarginal setae S1 (Fig. 23); [abdominal tergites in females without any patch (Fig. 19)]	***E. xerophilus* sp. nov**.

#### Eremiothrips brunneus

Taxon classificationAnimaliaThysanopteraThripidae

(zur Strassen)

D36221B3-1023-55AD-A030-81CB6E3C1D30

Figs 1–4

Ascirtothrips brunneus zur Strassen, 1975: 263.

##### Description.

**Figures 1–6.**
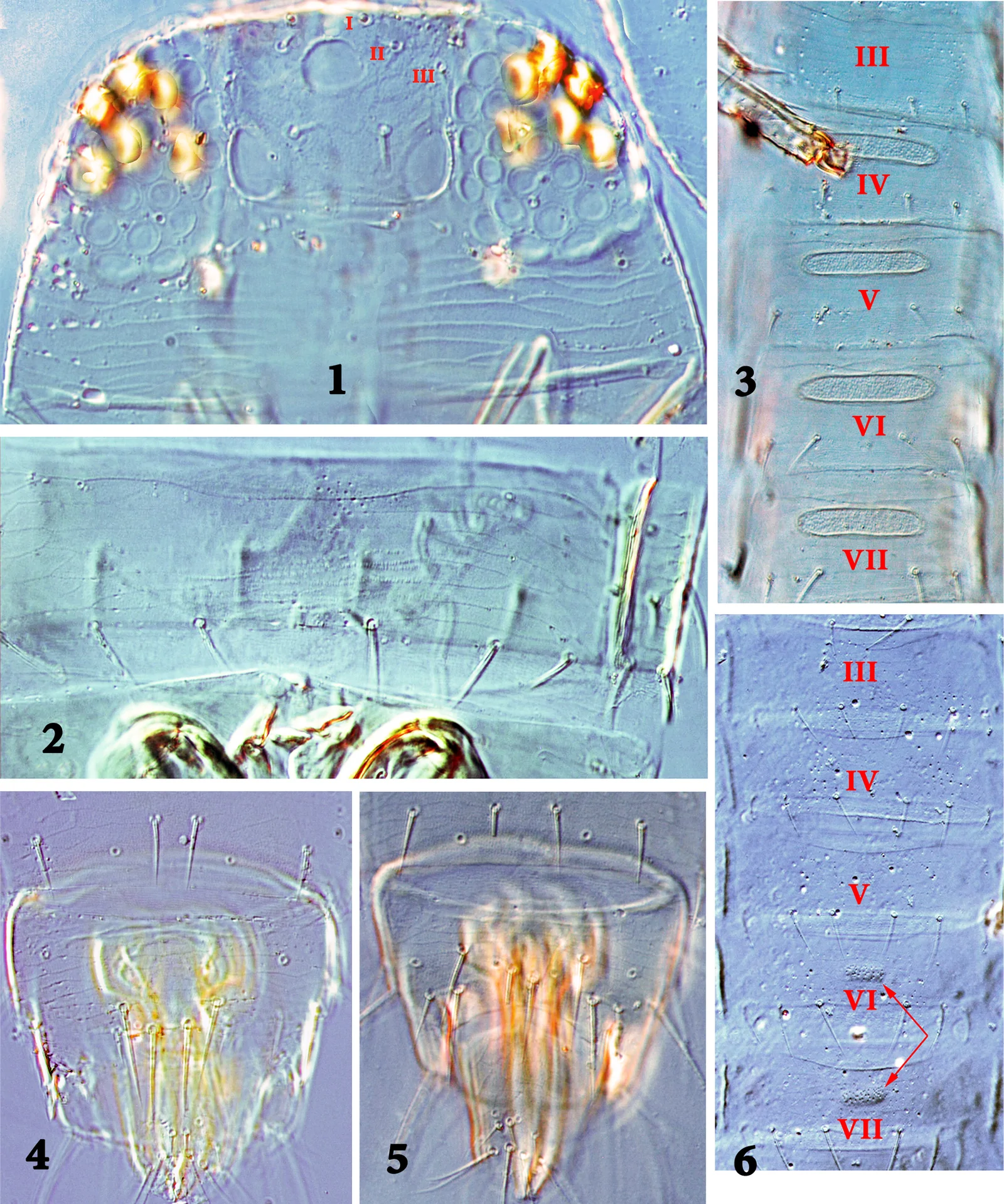
*Eremiothrips* spp. **1–4**. *Eremiothrips brunneus*. **1, 2**. Female: **1**. Head **2**. Sternum VII. **3, 4**. Male: **3**. Pore plates on sternites IV–VII; **4**. Tergites IX–X. **5, 6**. *Eremiothrips similis* male: **5**. Tergites IX–X; **6**. Pore plates on sternites VI–VII.

The species was described from Morocco based on females and was also reported from the Canary Islands (zur Strassen 1975). It is a unique species among its congeners based on the head with three pairs of anteocellar setae (Fig. 1). zur Strassen described the species based on a large series of females collected in southern Morocco during February 1965; however, no male was collected during the expedition (zur Strassen 1975). The species is newly recorded from Saudi Arabia, and the male is described for the first time. The females described from Morocco are largely brown, however, the population collected from the desert ecosystem of Saudi Arabia is shaded grey. Moreover, the placement of the S1 setae on sternum VII was not indicated in the zur Strassen’s description of the species. The type material present in the NHM and specimens collected from Saudi Arabia indicate that S1 setae on sternum VII are placed at the posterior margin. Usually, members of the genus *Eremiothrips* have these setae far ahead of the posterior margin on sternum VII (Bhatti 1972).

***Male macroptera***. Similar to female in general structure, smaller in size, unicolorous pale except terminal antennal segments VI–IX grey. Tergite IX without paired drepanae (Fig. 4). Sternites IV–VII with large transverse pore plates (Fig. 3).

***Measurements***. Body length 755. Head length 65, width 100, ocellar setae III 10. Pronotum length 75, width 125; posteroangular setae 12. Metanotal median setae 12. Fore wing length 400. Sternites III–VII pore plates width (length): IV 50 (15), V 50 (15), VI 50 (15), VII 47 (15). Antennal segments I–IX length: 15, 25, 30, 22, 25, 32, 10, 8, 12.

##### Material examined.

• Seven females and five males, **Saudi Arabia**, Riyadh, the Empty Quarter, Uruq bani ma’arid, 06.iv.2021, BT; • one female and one male, Uruq bani ma’arid, 05.vi.2021, BT, (deposited in KSMA).

#### Eremiothrips tetraenae sp. nov.

Taxon classificationAnimaliaThysanopteraThripidae

A7779717-FEB7-5286-9E4D-0788803D797A

https://zoobank.org/46008745-6E8D-43B6-96FC-B845C0F81635

Figs 7, 8, 9, 10, 11, 12, 13, 14, 15, 16, 17

##### Description.

***Female macroptera*** (Fig. 16). Body entirely pale including legs, except pterothorax and abdominal tergites shaded grey laterally; antennal segments I–II pale, III–V pale, except shaded grey at extreme apex, VI–IX grey. Fore wing pale with cilia grey and setae light brown.

**Figures 7–15.**
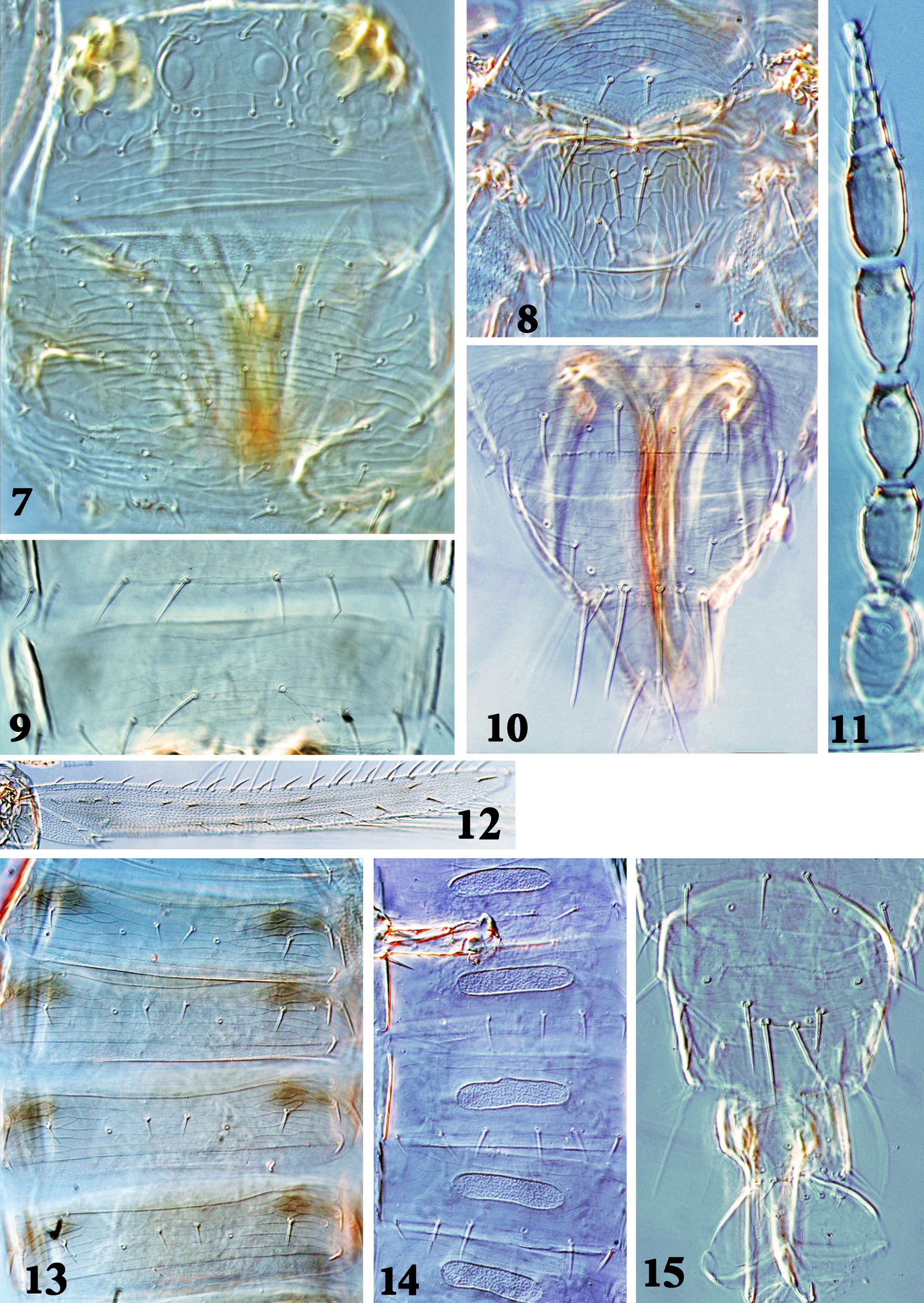
*Eremiothrips tetraenae* sp. nov. **7–13**. Female: **7**. Head and pronotum; **8**. Meso and metanotum; **9**. Sternite VII; **10**. Tergites VIII–X; **11**. Antennae; **12**. Fore wing; **13**. Tergites III–VI. **14, 15**. Male: **14**. Sternites III–VII **15**. Tergites IX–X.

Head (Fig. 7), wider than long; ocellar setae pair I in front of, and pair II lateral to fore ocellus; ocellar setae pair III positioned in front of hind ocelli within the triangle, smaller than the distance between their bases, and scarcely as long as longitudinal diameter of an ocellus; ocellar triangle smooth, without sculpture; occiput transversely striate (Fig. 7); mouth cone moderately long, extending across the middle of prosternum. Antennae 9-segmented, sense cones on segments III–IV forked; the inner sense cone on segment VI reaching the middle of segment VIII (Fig. 11). Pronotum (Fig. 7), wider than long, transversely striated with several discal setae; anterior margin with 5–6, and posterior margin with 3 pairs of setae as long as discal setae; one pair of pronotal posteroangular setae more than twice as long as posterior marginal setae. Mesonotum transversely striated, with one median and one lateral pair of setae, campaniform sensilla present; metanotum with equiangular reticles medially, and irregular longitudinal striae laterally; metanotal median setal pair arising behind anterior margin (Fig. 8). Fore wing first vein with 8–9 setae at basal, and 2–3 at distal half; second vein with about 7–8 setae (Fig. 15); posteromarginal cilia wavy.

Abdominal tergites with transverse sculpture laterally, and few striae medially; tergites II–IX with two pairs of campaniform sensilla, pair I present at anterior lateral margin, II posterolateral to the median pair of tergal setae (Fig. 12). Tergite VIII with a comb of sparse and fine microtrichia (Fig. 10); tergite IX setae S1 and S2 subequal in length; X without median dorsal split. Sternites smooth, with three pairs of posteromarginal setae; sternite VII with setae S1 arising in front of posterior margin (Fig. 9). Pleurotergites without discal setae.

***Measurements*** (holotype female). Body length 1025. Head length 90, width 150; ocellar setae III 10. Pronotum length 100, width 160; posteroangular setae 18. Metanotal median setae 18. Fore wing length 550. Tergite IX setae S1 55, S2 55. Antennal segments I– IX length: 15, 30, 35, 33, 33, 32, 10, 8, 15.

***Male macroptera*** (Fig. 17). Similar to female in general structure, smaller in size, unicolorous pale except terminal antennal segments VI–IX grey. Tergite IX without paired drepanae (Fig. 14). Sternites III–VII with large transverse pore plates (Fig. 13).

***Measurements*** (paratype male). Body length 790. Head length 75, width 125, ocellar setae III 10. Pronotum length 76, width 150; posteroangular setae 15. Metanotal median setae 15. Fore wing length 450. Sternites III–VII pore plates width (length): III 50 (15), IV 60 (15), V 60 (15), VI 55 (15), VII 50 (15). Antennal segments I–IX length: 15, 25, 35, 30, 30, 32, 10, 8, 13.

##### Etymology.

The species name is derived from the genus name *Tetraena*, referring to the plant from which the specimens were collected.

##### Material examined.

***Holotype*** female. **Saudi Arabia** • Jazan, Al Madaya, from leaves and flowers of *Tetraena simplex* [Zygophyllaceae], 22.ii.2024, 16°48'44.0"N, 42°42'01.3"E alt. 715 m, I. Rasool, (deposited in KSMA). ***Paratypes*** • 16 females, seven males were collected along with holotype.

##### Comments.

This new species is the second member of the genus that has pore plates on abdominal sternites III–VII, and lacks paired drepanae on abdominal segment IX. The other species that shares this condition is *E. bhattii*, but that is unique in having 8-segmented antennae, two pairs of pronotal posteroangular setae and oval to transverse pore plates (Minaei 2012). The new species described has 9-segmented antennae, one pair of posteroangular setae and transverse pore plates. This is the sixth member of the genus that lacks paired drepanae. The other four species are *E. bhattii*, *E. farsi*, *E. manolachei*, *E. similis* (Bhatti et al. 2003) and *E. brunneus*, from which it can be distinguished easily by the characters given in the key.

**Figures 16–17.**
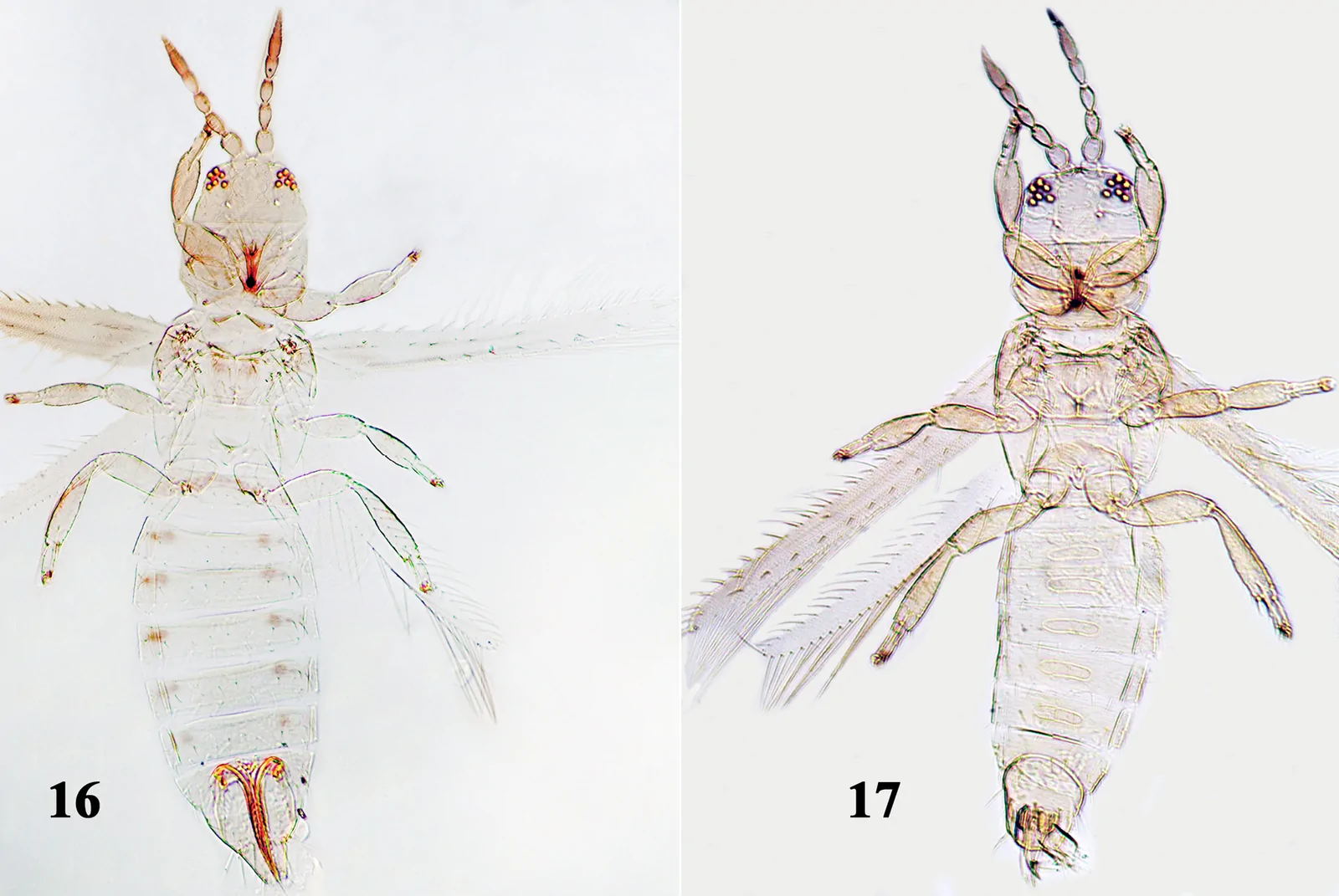
*Eremiothrips tetraenae* sp. nov. **16, 17**. Adults. **16**. Female; **17**. Male.

#### Eremiothrips xerophilus sp. nov.

Taxon classificationAnimaliaThysanopteraThripidae

1F311638-3360-5611-8696-4AC36EBA2024

https://zoobank.org/542EFC93-93E6-4739-97A7-DE61AAFF5892

Figs 16, 17, 18, 19, 20, 21, 22, 23, 24, 25, 26, 27

##### Description.

**Figures 18–25.**
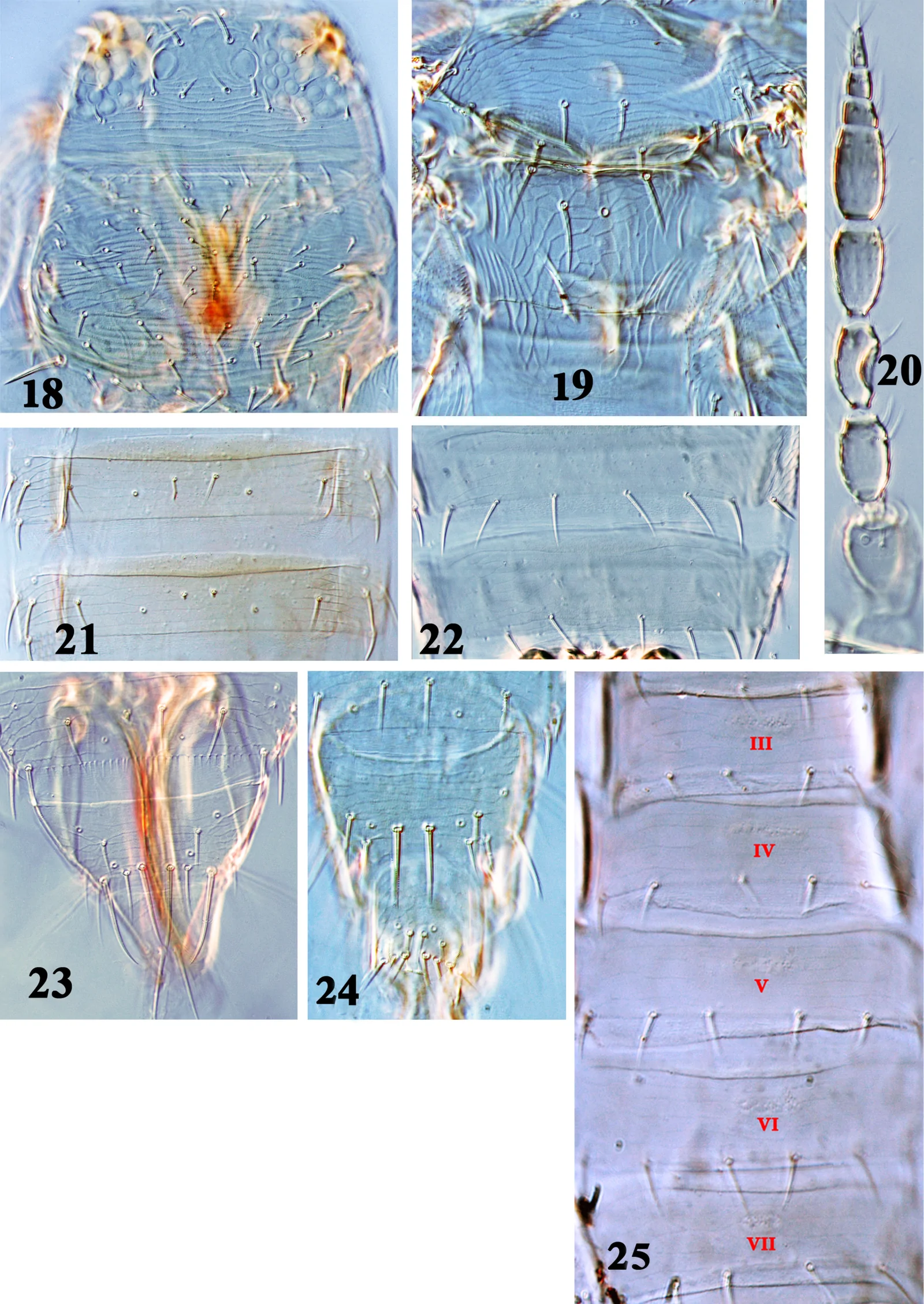
*Eremiothrips xerophilus* sp. nov. **18–23**. Female: **18**. Head and pronotum; **19**. Meso and metanotum; **20**. Antennae; **21**. Tergites V–VI; **22**. Sternites VI–VII; **23**. Tergites VIII–X. **24, 25**. Male: **24**. Tergites VIII–X; **25**. Pore plates on sternites III–VII.

**Figures 26–27.**
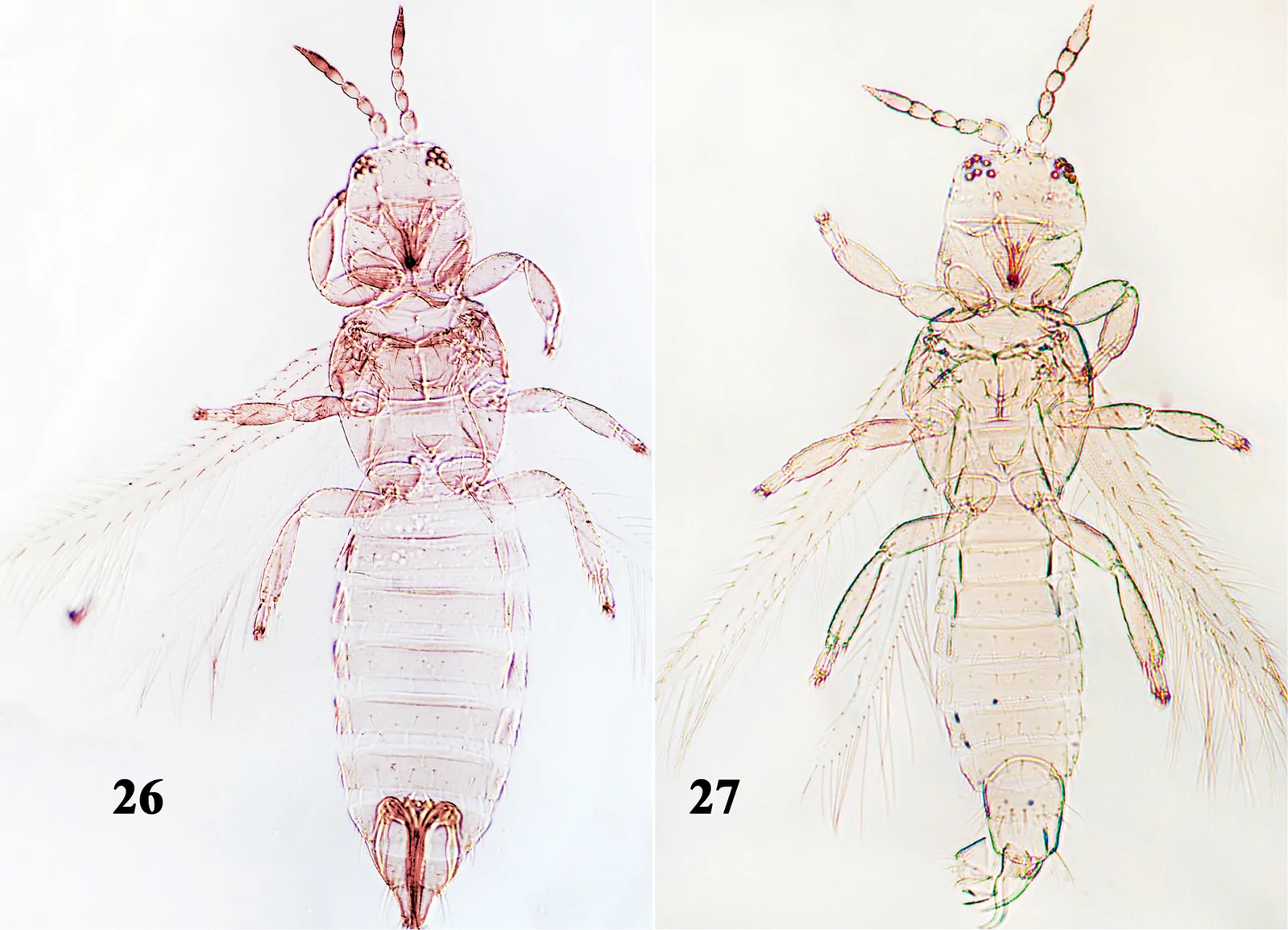
*Eremiothrips xerophilus* sp. nov. **26, 27**. Adults: **26**. Female **27**. Male.

***Female macroptera*** (Fig. 26). Body entirely pale including legs, antennal segments I pale, II grey, III–V pale, except shaded grey at extreme apex, VI pale at basal half, grey at distal half, VII–IX grey. Fore wings pale with cilia grey and setae light brown. Head (Fig. 16), wider than long; ocellar setae pair I in front of, and pair II lateral to the fore ocellus; ocellar setae pair III positioned in front of hind ocelli within the triangle, as long as or longer than the distance between their bases, and distinctly longer than diameter of an ocellus; ocellar triangle smooth, without sculpture; mouth cone moderately long, extending across the middle of prosternum. Antennae 9-segmented (Fig. 18), sense cones on segments III–IV forked; the inner sense cone on segment VI reaching to segment VIII. Pronotum (Fig. 16), wider than long, transversely striated with several discal setae; anterior margin with 5–6, and posterior margin with 4 pairs of setae; one pair of pronotal posteroangular setae more than twice as long as posterior marginal setae. Mesonotum transversely striated, with one median and one lateral pair of setae, with campaniform sensilla present; metanotum with longitudinal reticles; metanotal median setal pair arising behind anterior margin (Fig. 17). Fore wing first vein with 8–9 setae at basal, and 3–4 at distal half; second vein with about 11–12 setae; posteromarginal cilia wavy. Abdominal tergites (Fig. 19), with transverse striae laterally, and few weak striae medially; tergites II–IX with two pairs of campaniform sensilla, pair I present at anterior lateral margin, II posterolateral to the median pair of tergal setae. Tergite VIII with the comb of fine microtrichia (Fig. 21); tergite IX setae S1 and S2 equal or subequal in length; X with median dorsal split incomplete. Sternites smooth, with very weak striae and three pairs of posteromarginal setae; sternite VII with setae S1 arising in front of posterior margin (Fig. 20). Pleurotergites without discal setae.

***Measurements*** (holotype females). Body length 1130. Head length 95, width 180; ocellar setae III 25. Pronotum length 120, width 195; posteroangular setae 30. Metanotal median setae 28. Fore wing length 610. Tergite IX setae S1 50, S2 52. Antennal segments I– IX length: 16, 35, 35, 30, 30, 32, 10, 8, 15.

***Male macroptera*** (Fig. 27). Males are similar to females except smaller in size and paler in color. Tergite IX without paired drepanae (Fig. 22). Sternites III–VII with transverse and narrow pore plates, hardly visible (Fig. 23).

***Measurements*** (paratype males). Body length 700–840. Head length 72–80, width 125–140, ocellar setae III 18–20. Pronotum length 80–85, width 145–155; posteroangular setae 30–32. Metanotal median setae 25–27. Fore wing length 425–450. Sternites III–VII pore plates width (length): III 25 (5), IV 27 (5), V 25 (5), VI 25 (5), VII 17 (5). Antennal segments I–IX length: 15, 30–32, 28–30, 25–27, 25–27, 30–32, 10, 8, 13–15.

##### Etymology.

The species epithet *xerophilus* is derived from the Greek words “xēros”, meaning “dry,” and “phílos”, meaning “loving”. It refers to the species that is adapted to dry or arid environments in the country.

##### Material examined.

***Holotype*** female. **Saudi Arabia** • Al Jouf, Qariyat, collected from *Tamarix* sp. [Tamaricaceae], 25.ix.2022, 31°12'01.2"N, 37°34'56.4"E alt. 517 m, I. Rasool. ***Paratypes*** • three males, same data as holotype; • Madinah, Khayber, Al Taloh, three females, nine males, collected from *Tamarix* sp. [Tamaricaceae], 22.viii.2022; deposited in KSMA.

##### Comments.

This new species is the third member of the genus with pore plates on abdominal sternites III–VII and no paired drepanae on tergite IX. The other two species with this condition are *E. bhattii*, and *E. tetraenae* sp. nov. However, the former species is unique in having 8-segmented antennae, two pairs of pronotal posteroangular setae and oval to transverse pore plates (Minaei 2012). The new species is most similar to *E. tetraenae* sp. nov. described above in sharing 9-segmented antennae, one pair of posterior angular setae and transverse pore plates, but can be distinguished easily by the characters given in the key.

## Supplementary Material

XML Treatment for Eremiothrips brunneus

XML Treatment for Eremiothrips tetraenae

XML Treatment for Eremiothrips xerophilus

## Additional information

### Artificial Intelligence (AI) use

The authors accept full responsibility for the content of the manuscript, including the disclosure of any use of AI.

No AI tools were used in the preparation of this manuscript.

### Author ORCIDs

I. Rasool https://orcid.org/0000-0002-8955-2340

H.M. Aldhafer https://orcid.org/0000-0002-4911-2332

### Data availability

All of the data that support the findings of this study are available in the main text.
